# Encapsulation of Gold Nanostructures and Oil-in-Water Nanocarriers in Microgels with Biomedical Potential

**DOI:** 10.3390/molecules23051208

**Published:** 2018-05-18

**Authors:** Mariela Inostroza-Riquelme, Andrea Vivanco, Pablo Lara, Simón Guerrero, Edison Salas-Huenuleo, Alejandro Chamorro, Lisette Leyton, Karen Bolaños, Eyleen Araya, Andrew F. G. Quest, Marcelo J. Kogan, Felipe Oyarzun-Ampuero

**Affiliations:** 1 Advanced Center of Chronic Diseases (ACCDiS), Universidad de Chile. Santos Dumont 964, Independencia, Santiago 8380494, Chile; mariela.inostroza@postqyf.uchile.cl (M.I.-R.); andre.vivanco.p@gmail.com (A.V.); pablolara07@ug.uchile.cl (P.L.); simon.guerrero@um.uchile.cl (S.G.); edison.salash@gmail.com (E.S.-H.); achamorro.vet@gmail.com (A.C.); lleyton@med.uchile.cl (L.L.); eyleen.araya@unab.cl (E.A.); aquest@med.uchile.cl (A.F.G.Q.); 2 Departamento de Ciencias y Tecnología Farmacéuticas, Facultad de Ciencias Químicas y Farmacéuticas, Universidad de Chile, Santos Dumont 964, Independencia, Santiago 8380494, Chile; 3 Departamento de Química Farmacológica y Toxicológica, Facultad de Ciencias Químicas y Farmacéuticas, Universidad de Chile, Santos Dumont 964, Independencia, Santiago 8380494, Chile; 4 Cellular Communication Laboratory, Programa de Biología Celular y Molecular, Instituto de Ciencias Biomédicas, Facultad de Medicina, Universidad de Chile, Av. Independencia 1027, Independencia, Santiago 8380453, Chile; 5 Center for studies on Exercise, Metabolism and Cancer (CEMC), Universidad de Chile, Av. Independencia 1027, Independencia, Santiago 8380453, Chile; 6 Departamento de Ciencias Quimicas, Facultad de Ciencias Exactas, Universidad Andres Bello, Av. Republica 275, Santiago 8370251, Chile; karenlb1996@gmail.com

**Keywords:** gold nanoparticles, gold nanorods, nanoemulsions, curcumin, microgels, encapsulation

## Abstract

Here we report the incorporation of gold nanostructures (nanospheres or nanorods, functionalized with carboxylate-end PEG) and curcumin oil-in-water (O/W) nanoemulsions (CurNem) into alginate microgels using the dripping technique. While gold nanostructures are promising nanomaterials for photothermal therapy applications, CurNem possess important pharmacological activities as reported here. In this sense, we evaluated the effect of CurNem on cell viability of both cancerous and non-cancerous cell lines (AGS and HEK293T, respectively), demonstrating preferential toxicity in cancer cells and safety for the non-cancerous cells. After incorporating gold nanostructures and CurNem together into the microgels, microstructures with diameters of 220 and 540 µm were obtained. When stimulating microgels with a laser, the plasmon effect promoted a significant rise in the temperature of the medium; the temperature increase was higher for those containing gold nanorods (11–12 °C) than nanospheres (1–2 °C). Interestingly, the incorporation of both nanosystems in the microgels maintains the photothermal properties of the gold nanostructures unmodified and retains with high efficiency the curcumin nanocarriers. We conclude that these results will be of interest to design hydrogel formulations with therapeutic applications.

## 1. Introduction

Gold nanoparticles (Au-NPs) are nanomaterials that have received much attention due to their physical and chemical properties that make them particularly attractive for biomedical applications [1]. When Au-NPs are irradiated at a specific wavelength, local energy is released as heat, a Surface Plasmon Resonance (SPR) phenomenon referred to as the “photothermal effect” [2,3,4,5]. Due to this effect, gold nanostructures have the potential to kill cancer cells, to disaggregate amyloid plaques in Alzheimer disease, and also to release molecules in a spatio-temporal manner [6,7,8]. Notably, there are promising clinical trials in progress for Au-NPs [9]. However, it is necessary to consider the potential risks in the use of these nanomaterials for future clinical applications.

The most commonly used gold nanostructures are nanospheres (GNS), with an SPR absorption peak around 510 and 540 nm [2,10]. In terms of biological applications, gold nanostructures with the plasmon absorption in the NIR region, also called “NIR window” or “transparent region” (between 700 and 1100 nm) often are preferable, because the radiation of longer wavelength can penetrate human tissues without causing any damage to the organism [3,4]. Interestingly, gold nanorods (GNR) can be synthesized in a manner that permits adjusting their SPR to the required biological NIR window [3,4,6]. A classic example of a material used in this context is polyethylene glycol (PEG), which increases the stability of the nanostructures in several media [11,12].

Curcumin, the main pigment extracted from Curcuma longa L. (Zingiberaceae), has been widely studied for the treatment of cancer, in cell lines [13,14,15,16] and in patients [17,18]. Despite displaying encouraging effects, the use of curcumin is challenging due to its low solubility in aqueous phase (solubility of <0.5 µg/mL at pH 7.4) and high instability under biologically relevant conditions (pH, light, temperature and oxygen, among others) [16,19]. Vehiculization in oil-in-water (O/W) nanocarriers, as nanoemulsions, has emerged among the interesting strategies used to provide compatibility (solubility or dispersibility) between lipophilic molecules (such as curcumin) and aqueous media [20,21]. Interestingly, several studies have also demonstrated that lipophilic and unstable molecules remain stable within these vehicles, even after being exposed to harsh conditions [19,22,23].

Alginate is a polymeric material commonly used in drug delivery to improve the biocompatibility and stability of drugs. This polymer also has the ability to form gels and hydrophilic structures can be directly included in alginate microgels by mixing the polymeric solution with the selected hydrophilic compound. [24,25,26,27] Although, it is more difficult to include hydrophobic molecules in such microgels, the use of the previously described O/W nanocarriers allows compatibility of lipophilic molecules within aqueous solvents [20,21,23].

Thus here, we set out to include both gold nanostructures and curcumin in alginate microgels and to evaluate the effect of these microgels after stimulating them with a laser. We show that gold nanoparticles and O/W nanoemulsions can be incorporated into alginate microgels. The main variables evaluated were the geometry of gold nanostructures (nanospheres and nanorods) and the size of the microgels (220 and 540 µm). Also, in vitro experiments analyzed curcumin efficacy and safety using cancer (AGS) and non-cancer cells (HEK293T). Finally, the increase in temperature following irradiation was analyzed, after the encapsulation of both nanostructures (gold nanostructures and curcumin-loaded O/W nanoemulsion) in microgels.

## 2. Results and Discussion

### 2.1. Synthesis and Characterization of Gold Nanostructures

Gold nanostructures were synthesized with the aim of obtaining two different geometries (spheres and rods), and both were functionalized using polyethylene glycol with a carboxyl-end group (PEG-COOH). These PEG functionalized gold nanostructures were characterized by different techniques, including UV-vis-NIR spectrophotometry, dynamic light scattering (DLS) and laser Doppler anemometry (LDA). GNS-PEG-COOH exhibited the plasmon band at 521 nm while GNR-PEG-COOH exhibited two plasmon bands, at 524 and 754 nm, corresponding to transversal and longitudinal bands, respectively (see Figure S1 in the Supplementary Materials section). The obtained surface plasmon bands for GNS and GNR are characteristic of such systems and have been reported in the literature [2,3,5,7]. Also, the concentration of nanostructures determined by analyzing the UV-vis-NIR spectrum, was found to be 5.4 nM and 0.2 nM for GNS-PEG-COOH and GNR-PEG-COOH, respectively. According to the DLS characterization, the hydrodynamic diameter of GNS-PEG-COOH was 22.7 ± 7.6 nm with a poly-dispersion index (PDI) of 0.38 and a zeta potential of −19.7 ± 1.6 mV. On the other hand, two peaks of size, 4.2 ± 1.9 and 80 ± 41.7 nm were detected for GNR-PEG-COOH with a PDI of 0.57. The two peaks observed for GNR-PEG-COOH are due to differences in the longitudinal and transversal diameters, a common trait of these structures, as well as the PDI value, since the methodology employed to obtain the DLS values assumes that the nanostructures are spheroids and excludes particles with other geometries [4,6]. The zeta potential of these GNR-PEG-COOH was −16.5 ± 7.13 mV.

As reported, the functionalization of gold nanostructures with PEG can decrease their cytotoxicity, especially in the case of GNR, whose synthesis includes CTAB, a toxic component located on the surface of the nanoparticle [5,6]. In this respect, Almada et al. reported a significant decrease in the cytotoxicity of gold nanorods when functionalized using PEG and chitosan [6]. In addition, such functionalization prevents the nonspecific adsorption of biomolecules to nanorods, thus increasing their circulation time and providing more stability under storage conditions [8,11]. Additionally, the carboxyl groups improve the compatibility of the systems with the negatively charged polymeric solution required to develop alginate microgels, as we previously described [28].

**Figure 1:**
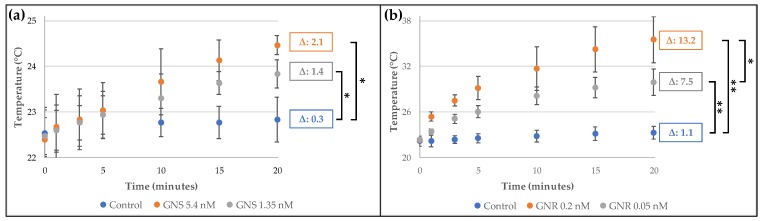
Temperature reached by gold nanostructures after irradiation for 20 min: (**a**) GNS-PEG-COOH irradiated at 532 nm and 50 mW power; (**b**) GNR-PEG-COOH irradiated at 808 nm and 350 mW power. Control: Milli-Q water. In both cases, Δ values were calculated by comparing with the initial average temperature (*n* = 3, * *p* < 0.05; ** *p* < 0.001).

Moreover, we determined the temperature changes produced by the photothermal effect when gold nanostructures were irradiated with a laser, a characteristic that is very relevant for future applications in therapy. For the irradiation, we chose the lasers with wavelengths centered near the plasmon bands (532 and 808 nm for GNS and GNR, respectively) [8]. As can be seen in Figure 1a, after irradiating GNS (5.4 nM, equivalent to 55 µg of gold/mL), a temperature increase of 2.1 °C was detected in the medium (a highly diluted suspension of GNS in water); and when additional dilutions were applied (4-fold dilution), the temperature rise was 1.4 °C. For GNR (Figure 1b), the increase in temperature was higher than for GNS (13.2 and 7.5 °C), even though the GNR concentration was lower (we tested 0.2 and 0.05 nM of GNR, equivalent to 14 µg of gold/mL and 0.28 µg of gold/mL, respectively). Although the concentration of GNS was approximately 4 times higher than the concentration of GNR, it is necessary to state that the power used for both lasers is different (i.e., for the 532 nm wavelength the power was 50 mW and for 808 nm the power was 350 mW). Therefore, in agreement with previous reports [7,10,29], the release of heat for the GNR is higher than to GNS at the concentrations and power analyzed.

### 2.2. Development and Characterization of Curcumin-Loaded O/W Nanoemulsions (CurNem), and Viability Assays

**Figure 2:**
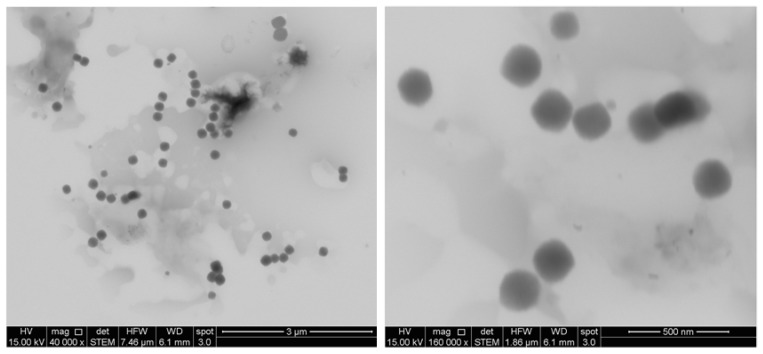
Electronic microscopy images of CurNem obtained at magnifications of 40,000 (**left**) and 160,000 (**right**).

Curcumin nanocarriers (CurNem) were prepared by the addition of an organic phase (that includes the curcumin) into water, and then by elimination of the organic solvents by rotatory evaporation [20]. The size of the obtained CurNem was 175.4 ± 11.0 nm, with low PDI (0.1–0.2), which is common for nanocarriers produced using this strategy [20,21]. The nanoemulsions have a negative zeta potential of −45.1 ± 6.5 mV, due to the use of Epikuron^®^, a mixture of surfactants including zwitterionic phospholipids, anionic fatty acids, phosphatidic acid, among others. Characterization by electron microscopy revealed such nanocarriers to be monodispersed, round and highly regular in shape (Figure 2), which correlates with the size-data obtained by dynamic light scattering. There are some reports indicating that curcumin-loaded nanoemulsions are smaller (15–140 nm), presumably because the elaboration methods employed include higher temperatures, sonication and homogenization [30,31]. On the contrary, in our method practically no additional energy input is required. The association efficiency of curcumin in our formulations was high (99.9 ± 0.1%) and the drug loading was equivalent to 2.1 ± 0.1%.

**Figure 3:**
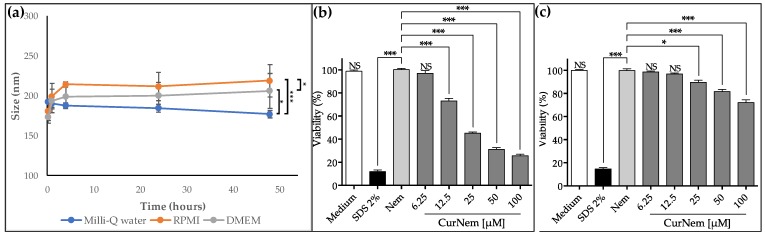
(**a**) Size of CurNem after incubation for 48 h at 37 °C in water, RPMI and DMEM (both culture medium enriched with 10% of FBS); (**b**) Viability of human gastric cancer cells (AGS) after 24 h of treatment with Nem and CurNem formulations; (**c**) Viability of human embryonic kidney cells (HEK293T) after 24 h of treatment with Nem and CurNem formulations (*n* = 3, * *p* < 0.05; *** *p* < 0.001, NS: No significant differences).

We also evaluated the in vitro efficacy of the curcumin nanocarriers against human gastric adenocarcinoma cells (AGS), as well as the safety with respect to non-cancerous human embryonic kidney cells (HEK293T) cells. First, we evaluated the stability of the formulation in the corresponding cell culture media (RPMI and DMEM, for AGS and HEK293T cells, respectively, both enriched with 10% of FBS). As shown in Figure 3a, formulations were stable for at least 48 h. Also, only a minor increase in the hydrodynamic size (25–40 nm) with similarly low polydispersity values (<0.2) was observed, confirming the stability of the nanocarriers. In Figure 3b, the results obtained in viability assays using AGS cells are shown. Nem vehicles (blank nanoemulsions) did not affect cell viability, which is attributable to the specific components utilized, all of which are FDA approved. Importantly, the results indicate that CurNem diminished cell proliferation. With doses as low as 12.5 µM of curcumin, significant decrease in viability is detected. Cell viability decreased to 26% of control values when 100 µM of the formulation was administered (IC_50_ of 24.5 µM). Considering that curcumin is a lipophilic molecule (log P 2.3—2.6), it is expected to be retained mainly entrapped in the oily nucleus of nanoemulsions, when dispersed in aqueous media, as demonstrated by Guan et al. [32]. Results shown in Figure 3b indicate that CurNem is also effective at killing cancer cells. Given that many of the anticancer effects attributed to Cur are related to intracellular phenomena [33,34,35,36], we suspect that effective cellular uptake and internalization of the formulations is relevant and facilitated by the size [37] and the inherent permeability enhancing properties of phospholipids (main component of the surfactant) employed [38]. Preliminary assays conducted by us, indicate that the endosomal entry route is favored by using our formulation, which is consistent with other studies testing different nanoemulsions [32]. A report is available evaluating cytotoxicity in prostate cancer cells (PC-3 cells, cultured in RPMI enriched with 10% FBS) after the administration of curcumin nanoemulsions (35 nm in size), produced with a medium chain triglyceride, cremophor RH40, glycerol and water [32]. Despite the differences in how formulations were prepared and the cells tested (culture medium is the same), the obtained results after 24 h appear similar to ours. Unfortunately, the authors did not include information on IC_50_ values [32].

In order to further analyze the safety of our formulations, cell viability was also evaluated following the application of CurNem to HEK293T cells. At the highest doses tested, CurNem diminished cell viability by only 28% (Figure 3c). These results are encouraging and demonstrate that the formulation is more effective in decreasing the viability of the cancer cells tested (AGS) than in non-cancerous cells (HEK293T).

### 2.3. Development and Characterization of Alginate Microgels Loaded with Gold Nanostructures and CurNem

**Figure 4:**
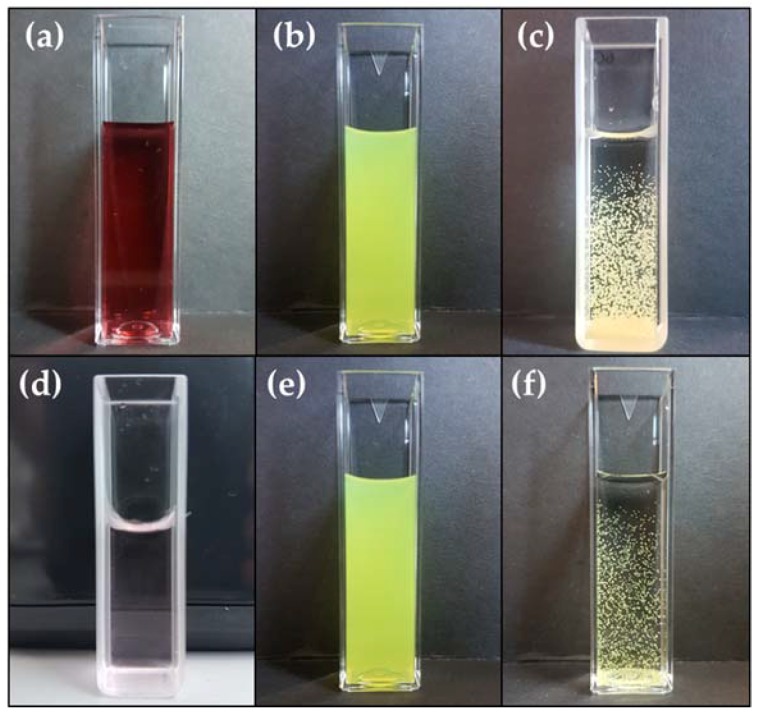
Optical image of the nanosystems (gold nanostructures and CurNem) and the microgels containing them. Upper panels: (**a**) GNS (5.4 nM of nanospheres); (**b**) CurNem (1.5 mM of curcumin) and (**c**) Microgels (≈220 µm) containing GNS (1.35 nM of nanospheres) and CurNem (375 µM of curcumin). Lower panels: (**d**) GNR (0.2 nM of nanorods); (**e**) CurNem (1.5 mM of curcumin) and (**f**) Microgels (≈220 µm) containing GNR (0.05 nM of nanorods) and CurNem (375 µM of curcumin).

Alginate microgels were produced using the dripping strategy, which involves adding dropwise an alginate solution that also contains the gold nanostructures and CurNem to a calcium solution. It is important to note that both nanostructures are ionically compatible with the negatively charged alginate. In this case, both have negative surface charges, as evidenced by the negative zeta potential, which guarantees compatibility of the surface charges [28]. The homogenous polymeric dispersion (containing the nanostructures) was added dropwise to a calcium solution using automatized equipment (Encapsulator B-395 Pro, Büchi, Flawil, Switzerland) that permitted generating microgels of two different sizes (using two different nozzles). In Figure 4, the different combinations of components that permitted obtaining microgels (gold nanostructures, CurNem and alginate containing them in proportions of 1:1:2, respectively) are shown.

Microgels containing the gold nanostructures and CurNem were spherical, and, as shown in Figure 4c,f, the formulations possessed the same color as the encapsulated nanosystems. The sizes of the microgels were studied by optical microscopy and found to depend on the nozzle used. Microgels produced using a 150 µm nozzle had diameters of 220 ± 42 µm, while those microsystems prepared with a 300 µm nozzle were 540 ± 98 µm in diameter.

One of our objectives here was to determine whether gold nanostructures maintained the ability to increase the temperature after stimulation with a laser, once they had been encapsulated in alginate microgels containing CurNem. It was also of interest to determine if the microgels maintained their stability once the temperature increased following laser stimulation.

**Figure 5:**
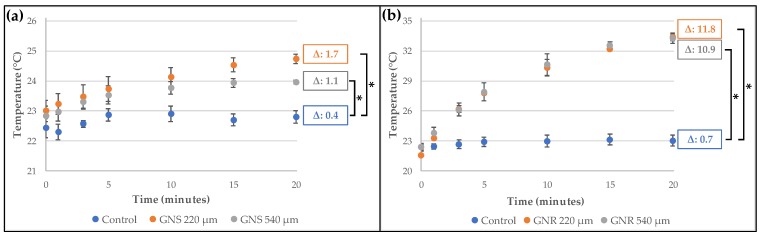
Temperature reached by microgels (220 and 540 µm of diameter) containing CurNem and gold nanostructures after irradiation for 20 min: (**a**) GNS 1.35 nM (532 nm laser and 50 mW power); (**b**) GNR 0.05 nM (808 nm laser and 350 mW power). Control corresponds to Milli-Q water and in both cases, the Δ values were calculated by comparing with the initial average temperature (*n* = 3, * *p* < 0.001).

Figure 5 shows the temperature reached by microgels once they were submitted to laser stimulation. As shown in Figure 5a, microgels of 220 µm increased the temperature by 1.7 °C, while an increase by 1.1 °C was recorded for 540 µm microgels, in both cases loaded with GNS. In Figure 5b, the temperature increases for microgels loaded with GNR are shown after the irradiation. In this case, increases of 11.8 °C and 10.9 °C were observed for 220 µm and 540 µm microgels, respectively. In both cases (microgels containing GNS and GNR), there were no significant differences in the final temperature, when comparing the two microgels of different sizes with non-encapsulated gold nanostrucutures (at the same concentration, see Figure 1).

The difference in the final temperature observed for GNS and GNR is attributable to the well-established capacity of nanorods to generate higher temperatures than nanospheres after being stimulated [10,29]. This was also confirmed by our results shown in Figure 1, where non-encapsulated GNR generates a greater increase in temperature than the GNS.

To the best of our knowledge, there are no other studies available that evaluate the concomitant encapsulation of gold nanoparticles and O/W nanoemulsions in microgels, and the effects of subsequent stimulation with a laser. Given that the temperature generated by the gold nanostructures, encapsulated or not in the microgels, were similar, a wide range of possibilities can be envisioned considering the combination of these components for the design of formulations with therapeutic potential.

**Figure 6:**
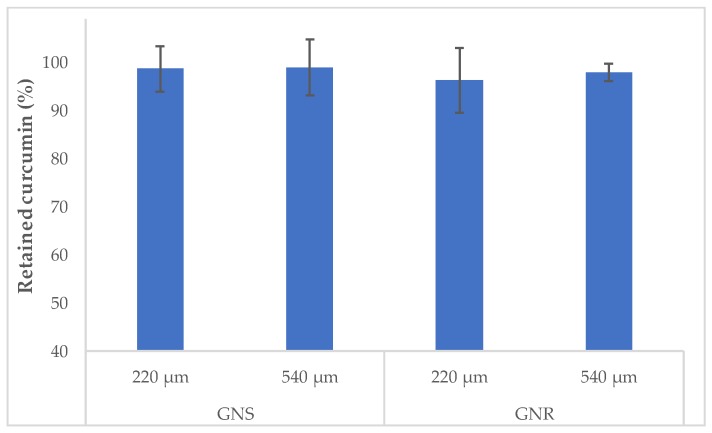
Percentage of retained curcumin in microgels loaded with CurNem and gold nanostructures after irradiation for 20 min.

In order to determine if microgels retained the nanocarriers containing curcumin after the irradiation stimulus, we analyzed the content of curcumin. Importantly, we were unable to detect curcumin in the supernatant medium after the irradiation. Additionally, a direct methodology (dissolving the microgels and analyzing the inner content), confirms that curcumin remained almost completely encapsulated in the microgels after the irradiation (98.0 ± 4.8%, Figure 6).

The high degree of encapsulation maintained following irradiation is likely due to the fact that the temperatures reached by the gold nanostructures were not sufficient to produce significant conformational changes in the bulk alginate matrix of the microgel, which would lead to release of the encapsulated nanosystem. Given that the methodology described in this paper is simple, we propose the use of thermosensitive polymers such as poly *N*-isopropylacrylamide (PNIPAm) that can be incorporated into the alginate microgels by either physical or chemical interactions [39,40]. The lower critical solution temperature (LCST) of PNIPAm (defined as the temperature at which the polymer transits from a soluble to a shrunken state) can be adjusted to temperatures that are slightly higher than the body temperature (38–40 °C). Such systems should remain intact within the human body, but then can be expected to release the nanocarrier containing the active molecule(s), once the stimulus is applied (for example after laser stimulation). This strategy is favored over generating higher temperature into microgels (increasing the concentration of gold nanostructures, for example), bearing in mind the high sensitivity of curcumin to heat [31,41].

## 3. Materials and Methods

### 3.1. Materials

All reagents and solvents were analysis grade, used without further purification. Gold nanostructures synthesis: tetrachloroauric acid, dehydrated tribasic sodium citrate, cetyltrimethyl-ammonium bromide (CTAB), sodium borohydride, ascorbic acid and silver nitrate were purchased from Sigma-Aldrich (St. Louis, MO, USA); hydrochloric acid fuming (Merck, Darmstadt, HE, Germany). mPEG-SH and SH-PEG-COOH (5 kDa, JenKem Technology, Beijing, China). Curcumin nanoemulsions: curcumin (from Curcuma longa, C1386, Sigma-Aldrich), Epikuron 145V (Cargill, Barcelona, Spain), Miglyol 812 (Sasol GmbH, Hamburg, Germany), acetone and ethanol (Merck). Microgels: low viscosity sodium alginate (Sigma-Aldrich), anhydrous calcium chloride (Merck). Milli-Q water used in all the experiments was obtained from the purification of distilled water with a Simplicity SIMS 00001 equipment (Millipore, Molsheim, France). Cell culture: RPMI 1640, DMEM high glucose medium, penicillin and streptomycin (Gibco-BRL, Paisley, UK), fetal bovine serum (FBS, Biological Industries, Cromwell, CT, USA). Viability assays: 3-(4,5-dimetylthiazol-2-yl)-5-(3-carboxymethoxyphenyl)-2-(4-sulfophenyl)-2H tetrazolium inner salt (MTS) proliferation assay kit (Promega, Madison, WI, USA).

### 3.2. Synthesis and Characterization of Gold Nanostructures

Gold nanospheres (GNS) and Nanorods (GNR) were synthesized and surface modified with a carboxyl-end polyethylene glycol (PEG-COOH), according to the following protocols:

#### 3.2.1. Synthesis and Characterization of Gold Nanospheres (GNS)

GNS were synthetized by citrate reduction of HAuCl_4_ according the Turkevich protocol [2]. Briefly, a solution of HAuCl_4_ 100 mM was refluxed for 15 min, and then a warm solution of sodium citrate 38.8 mM was quickly added. The reflux was maintained for 30 min until a deep red suspension of GNS was obtained. This suspension was filtrated using a 0.45 µm syringe filter and stored at 4 °C.

To functionalize the GNS with a surface carboxyl group, 5 nM of uncoated GNS were incubated by mixing with an aqueous solution of 1 mg/mL of SH-PEG-COOH in a volume ratio of 1:20 (an excess of PEG was added in order to ensure a homogeneous conjugation). GNS-PEG-COOH spheres were purified using a 0.45 µm syringe filter, followed by three steps of centrifugation (16,100× *g* × 30 min each) and reconstitution of the pellet in Milli-Q water.

The obtained nanostructures were characterized by UV-Vis spectrophotometry (Lambda 25 spectrophotometer, Perkin Elmer, Waltham, MA, USA) and the concentration of gold nanoparticles was determined as reported by Liu et al. [42]. The hydrodynamic diameter and polydispersity index of the nanostructures was characterized by dynamic light scattering (DLS), and the zeta potential determined by laser Doppler anemometry (LDA) in a ZetaSizer NanoZS (Malvern Instruments, Malvern, UK).

#### 3.2.2. Synthesis and Characterization of Gold Nanorods (GNR)

GNR were prepared using the seed mediated method [5]. To obtain the gold seeds, a reduction of 250 μL HAuCl_4_ 0.01 M with 600 μL of cold-prepared sodium borohydride 0.01 M in a matrix solution of 9.75 mL of CTAB 0.1 M was performed. Seeds were kept at 27 °C for 2 h before use. Then, a growth solution was prepared adding 500 μL of HAuCl_4_ 0.01 M, 75 μL of AgNO_3_, 55 μL of ascorbic acid 0.1 M, and 250 μL of HCl 0.1 M in a solution of CTAB 0.1 M. To start the growth process, 12 μL of the previously prepared seed solution were added to the growth solution, incubated for 10 min at 27 °C and centrifuged at 7030× *g* for 15 min, removing the supernatant and resuspending the pellet in Milli-Q water.

To functionalize the GNR with a surface carboxyl group, 50 µL mPEG-SH (1 mM) were added to 10 mL of GNR (1 nM) and stirred for 10 min. The GNR-PEG rods were centrifuged at 20,800× *g* for 10 min. The supernatant was discarded and the pellet was resuspended in 10 mL of Milli-Q water. Then, a solution of 300 µL of HOOCPEG-SH (1mM) was stirred with GNR-PEG for 1 hour, resulting in mixed coverage of mPEG-SH y HOOC-PEG-SH on the surface of the GNR. The functionalized nanorods were centrifuged twice at 20,800× *g* for 10 min. The supernatant was removed and the pellet resuspended in 10 mL of Milli-Q water.

GNR were characterized by vis-NIR absorption spectra (Lambda 25 spectrophotometer, Perkin Elmer). The concentration of gold nanorods was determined as reported by Adura and et al. [5]. The hydrodynamic diameter, polydispersity index and zeta potential were determined by DLS and LDA, respectively.

#### 3.2.3. Irradiation of Gold Nanostructures

**Figure 7:**
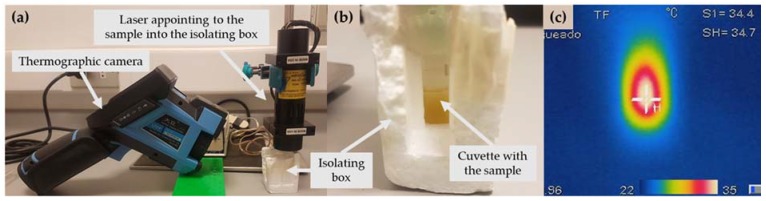
(**a**) Disposition of the sample (inside the isolating box), laser and thermographic camera; (**b**) Optical image of the sample inside the isolating box; (**c**) Thermographic image of the sample.

GNS-PEG-COOH and GNR-PEG-COOH were stimulated with a laser selected according to each plasmonic band. Thus, GNS were irradiated with a green laser LDCU5/9020 laser (Power Technology, Alexander, AR, USA) that emits a beam of light of 532 nm and a maximum power of 45.0 mW. On the other hand, GNR were stimulated with an IQ1A350 laser (Power Technology) with a beam of light of 808 nm and maximum power of 350 mW. In both cases, a power source with a potency of 2 mV was used and the laser was located in the superior extreme of the cuvette (4 centimeters under the sample, Figure 7a).

A volume of 500 µL of gold nanostructures was added to a polycarbonate cuvette of reduced volume (volume of cuvette: 1 mL). The experiments were done at room temperature (21–23 °C, registering the humidity and temperature, with an Indoor Thermomether Hygro and Clock (TH96, Qingdao TLead International Co., Qingdao, China) and using an isolating polystyrene box (3 × 4 × 6 centimeters) in order to avoid changes of temperature caused by the environment oscillation. The isolation box had two uncovered sides: the superior to allow the input of the laser beam, and the side to allow the temperature measuring, as showed in Figure 7b.

Each formulation was irradiated for 20 min and the temperature increase was monitored by infrared thermography, as shown in Figure 7c, using an infrared camera (DALI LT3, Zhejiang Technology, Hangzhou, China), located at 10 centimeters from the cuvette. The camera was set to measure and register the hottest point (emissivity index adjusted to 0.96). The control was 500 µL of Milli-Q water irradiated with each laser at the same conditions.

### 3.3. Elaboration of Curcumin-Loaded O/W Nanoemulsions (CurNem)

Nanoemulsions were produced using the solvent displacement method [20], consisting in adding an organic phase containing 125 μL of Miglyol 812 (a neutral oil formed by esters of caprylic and capric fatty acids and glycerol, density 0.945 gr/cm^3^), 30 mg of Epikuron 145 V (a phosphatidylcholine-enriched fraction of soybean lecithin), 2.76 mg of curcumin, 0.5 mL of ethanol and 9.5 mL of acetone; over an aqueous phase composed by Milli-Q water (20 mL). Once the nanoemulsion was instantaneously formed (evidenced by the milky appearance of the suspension), it was rotaevaporated until a volume of 5 mL with the aim of extracting the organic solvents from the mixture (ethanol and acetone) and concentrating the formulation until 1.5 mM of curcumin. Blank nanoemulsions (Nem) were developed using the same procedure but avoiding the addition of curcumin. The hydrodynamic diameter and zeta potential of the colloidal systems were determined by DLS and LDA.

Scanning transmission electron microscopy (STEM) analyses were done in an Inspect 50 microscope (FEI, Holland). STEM images were obtained by sticking a droplet (10 μL) of CurNem on a copper grid (200 mesh, covered with Formvar) for 2 min, then removing the droplet with filter paper avoiding the paper touching the grid, then washing twice the grid with a droplet of Milli-Q water for 1 min, and removing the droplet with filter paper. Later, the sample was stained with a solution of 1% phosphotungstic acid by sticking a droplet of this solution on the grid for 2 min and removing the droplet with filter paper. Finally, the grid was allowed to dry for at least 1 h before analysis.

The association efficiency of curcumin in the nanocarriers was determined by analyzing the difference between the total amount of curcumin in the final formulation and the free curcumin. As the excipients used to prepare the formulation are soluble in acetone (Epikuron 145 V, Miglyol 812 and curcumin), the total amount of curcumin was easily estimated by dissolving an aliquot of non-isolated curcumin-loaded nanocarriers with acetone (1 volume of the formulation and 2 volumes of acetone) and measuring the absorbance at 424 nm (Lambda 25 spectrophotometer, Perkin Elmer). The free curcumin was estimated by isolating an aliquot of the sample using Vivaspin^®^ tubes (MWCO 100 kDa, 8500 G × 20 min), and then dissolving the isolated curcumin in acetone to measure the absorbance. The standard curve of curcumin in acetone was linear (*r*^2^ > 0.999) in the range of concentrations between 1 and 6 mg/L (molar extinction coefficient was 63291 M^−1^cm^−1^).

The drug loading of CurNem was calculated by dividing the mass of curcumin contained in the nanoemulsions compared with the total weight of the formulation (determined after freeze-drying the complete formulation).

### 3.4. In Vitro Determination of Efficacy and Safety of CurNem

We also evaluated in vitro efficacy (on cancer cells) and safety (on non-cancerous cells) of CurNem, and for this aim we chose two representative cell lines: human gastric adenocarcinoma cells (AGS) and human embryonic kidney cells (HEK293T), respectively. We determined cell viability after 24 h using the MTS assay. Thus, prior to this assay, we analyzed the stability of CurNem in the corresponding cell culture mediums, corresponding to RPMI and DMEM, respectively.

#### 3.4.1. Stability of CurNem in Cell Culture Medium

To determine the stability of CurNem in the cell culture media, three aliquots of 10 µL of CurNem were diluted in 990 µL of Milli-Q water, or each culture medium (RPMI and DMEM both enriched with 10% of FBS) and incubated in an orbital shaker (LSI-3016R, LabTech, Daihan LabTech, Kyonggi-Do, Korea) at 37 °C for 48 h. Samples were taken at different times (0.25, 1, 4, 24 and 48 h). Then, size and polydispersity index of the nanosystems were determined by DLS.

#### 3.4.2. Cell Culture

Human gastric adenocarcinoma cells (AGS) were cultured in RPMI 1640 supplemented with 10% of FBS, 100 UI/mL penicillin and 100 µg/mL streptomycin, as described Urra et al. [43]. Human embryonic kidney cells (HEK293T, ATCC) were cultured in DMEM high glucose medium containing 10% of FBS, 100 UI/mL penicillin and 100 µg/mL streptomycin, as described by Torres et al. [44]. Both cells were cultured at 37 °C and 5% CO_2_.

#### 3.4.3. Viability Assays

Cells were seeded in 96-well plates at a density of 1 × 10^4^ cells/well and incubated for 24 h in culture medium. Then the medium was removed and replaced by 90 µL of fresh medium and 10 µL of each treatment and maintained for 24 h. The treatments involved adding CurNem at different concentrations (6.25, 12.5, 25, 50, and 100 µM of curcumin in each well of the microplate). As controls, the corresponding cell culture medium, a blank nanoemulsion (Nem) and SDS 2% (death control) were used. Cell viability was evaluated by measuring the mitochondrial activity using the MTS^®^ assay, according to the instructions of the manufacturer (Promega) as described previously Adura et al [5].

### 3.5. Development and Characterization of Alginate Microgels Loaded with Gold Nanostructures and CurNem

Alginate microgels were automatically produced using the dripping technique. A solution of low viscosity alginate (2% *w*/*v*) was mixed with CurNem (1.5 mM of curcumin) and gold nanostructures (5.4 nM of GNS or 0.2 nM of GNR, both functionalized with PEG-COOH), in proportion 2:1:1. This anionic suspension was pumped using a Microencapsulator B-395Pro (Büchi) over a stirring calcium chloride solution (2% *w*/*v*). To prepare the microgels two different nozzles were selected (150 and 300 µm), the set parameters in the microencapsulator depended on the nozzle used (flux of dripping: 2.5 and 6.5 mL/min, respectively; voltage: 900 and 1280 V, respectively and frequency: 6000 Hz in both cases). The excess of calcium chloride was extracted washing 3 times the microgels with Milli-Q water.

Size and shape of the microgels were analyzed by optical microscopy (Olympus CKX41, Arquimed, Tokyo, Japan) at 4x magnification, using a digital camera (Digital Sight DS-Fi2, Nikon, Tokyo, Japan) with the software Micrometrics SE Premium (Accu-Scope, Commack, NY, USA). The mean size and distribution was determined by analyzing the size of 80 microgels using the software ImageJ^®^ (National Institutes of Health (NIH), Bethesda, MD, USA).

#### Irradiation of Microgels Containing CurNem and Gold Nanostructures

A similar protocol to that described in Section 3.2.3. was used to irradiate the microgels samples. In this case, we used 500 µL of concentrated microgels (we previously marked the cuvette with 500 µL of water, and then we completed the volume with microgels, eliminating the excess of water), using the same polystyrene-isolating box showed in Figure 6b. The samples were irradiated for 20 min with a laser beam of 532 nm or 808 nm, depending on the contained gold nanostructures (GNS or GNR, respectively). The control was 500 µL of alginate microgels containing just CurNem and irradiated with each laser at the same conditions.

To evaluate the retained CurNem in the microgels after the irradiation, we slightly modified the previous protocol. This time, we used a cuvette with 500 µL of microgels and 500 µL of water; and after irradiating for 20 min we evaluated the supernatant in an UV-vis spectrophotometer without diluting the sample. Furthermore, we completely dissolved 500 µL of different samples (irradiated and non-irradiated microgels) in 2 mL of a solution of 55 mM sodium citrate, a final volume of 5 mL was achieved by adding Milli-Q water. We measured each sample in an UV-vis spectrophotometer to analyze the retained CurNem on the non-irradiated MGs, and it was compared with the quantity retained in the irradiated MGs.

### 3.6. Statistical Analysis

The statistical significance of the differences between formulations was determined by the application of two-way analysis of variance (ANOVA) followed by a two-sample t-test for equal or unequal variances. Differences were considered significant at *p* < 0.05.

## 4. Conclusions

We have used an efficient and simple strategy to encapsulate two different gold nanostructures (spheres and rods), both functionalized with a carboxyl-end PEG, along with negatively charged O/W curcumin nanocarriers, in alginate microgels. Curcumin was included in O/W nanoemulsions, and viability assays in both cancer and non-cancer cell lines (AGS and HEK293T, respectively) demonstrated a detrimental effect on AGS cells and relative safety for HEK293T cells. Alginate microgels were produced including gold nanostructures (GNS or GNR) and CurNem, resulting in microsystems of 220 and 540 µm. These microgels when stimulated with a laser lead to increments in temperature similar to non-encapsulated gold nanostructures. The curcumin nanocarrier remained encapsulated to over a 98% after irradiation. We postulate that the above results will be of importance to readers interested in designing hydrogel formulations that also could include thermosensitive polymers. In doing so, formulations for a controlled drug release, such as those modulated by laser stimulation would become available that permit the controlled release of active molecules.

## Supplementary Materials

The following are available online, Figure S1: UV-vis-NIR spectra of GNS-PEG-COOH and GNR-PEG-COOH.
