# Supplementary figures and images for: Encapsulation of Gold Nanostructures and Oil-in-Water Nanocarriers in Microgels with Biomedical Potential
Source: Molecules. 2018 May 18;23(5):1208. doi: 10.3390/molecules23051208 (PMC6099665; doi:10.3390/molecules23051208)

## Supplementary Materials

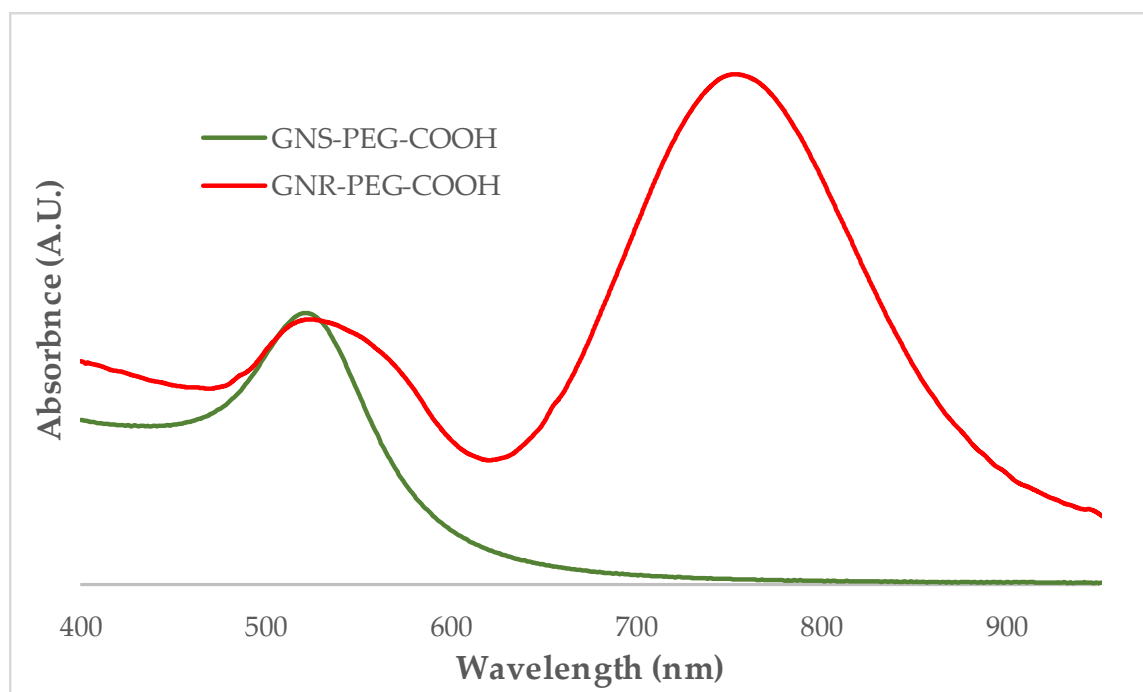

**Figure S1:** UV-vis-NIR spectra of GNS-PEG-COOH and GNR-PEG-COOH.

Supplement: Supplementary file 1 [file molecules-23-01208-s001.pdf]
